# Supplementary material for: Adenosine A2A Receptor Activation Regulates Niemann–Pick C1 Expression and Localization in Macrophages
Source: Curr Issues Mol Biol. 2023 Jun 7;45(6):4948–69. doi: 10.3390/cimb45060315 (PMC10297445; doi:10.3390/cimb45060315)
Supplement: Supplementary file 1 [file cimb-45-00315-s001.zip › cimb-2409538-supplementary.pdf]

Adenosine A_{2A} receptor activation regulates Niemann-Pick C1 expression and localization in macrophages

Supplementary Figures

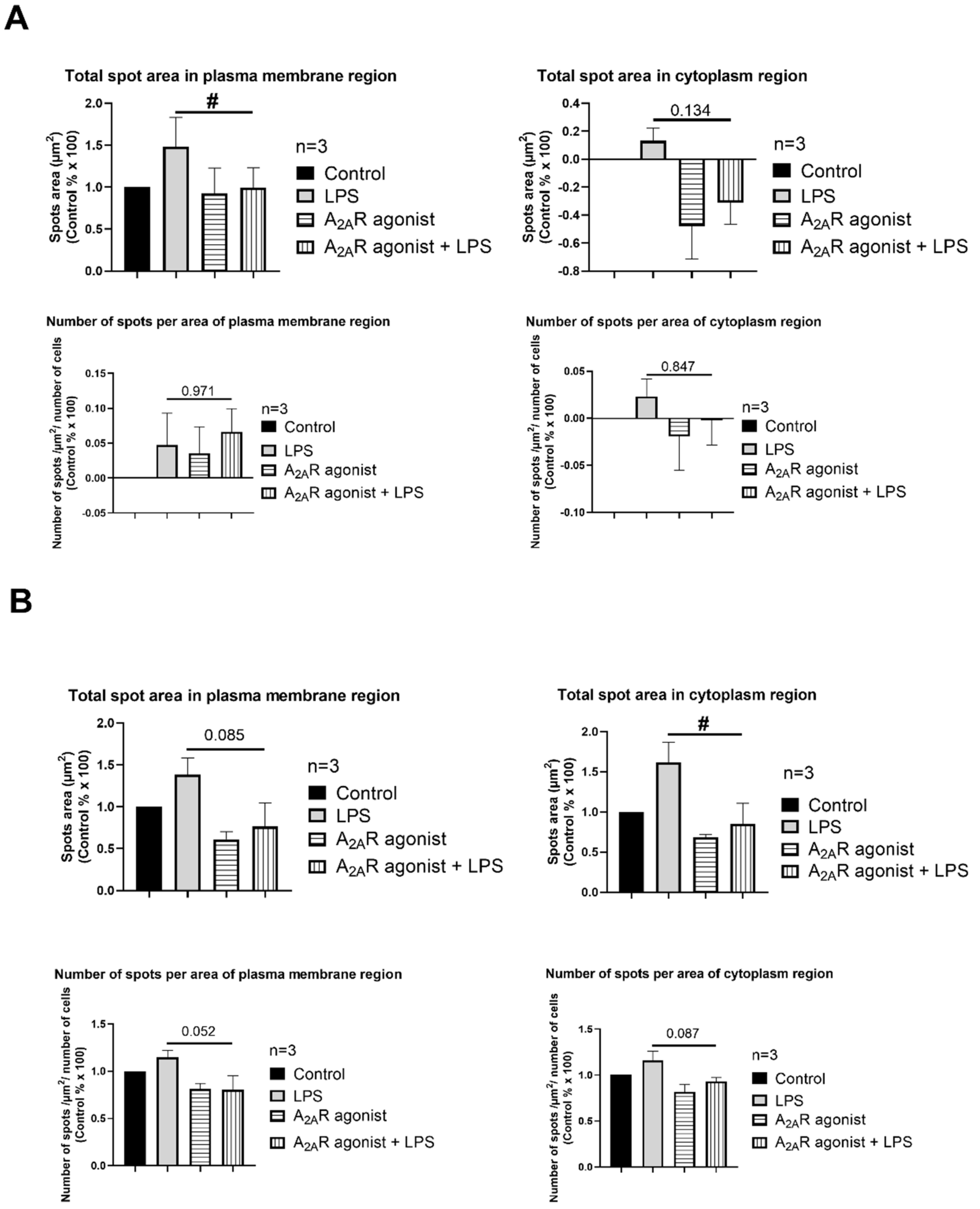

Figure S1. A_{2A}R activation decreases cell surface deposition of NPC1 in RAW 264.7 and mouse IPMΦs cells. Immunofluorescence staining of RAW 264.7 (A) and IPMΦ cells (B) were made using NPC1 specific, primary and Alexa-488 conjugated anti-rabbit secondary antibody. NPC1 specific fluorescence intensity was measured after LPS activation and treatment with the A_{2A}R agonist CGS21680. Cellular features such as total spots area and number of spots per area of membrane and cytoplasm regions were extracted. Data obtained from the individual analysis of 400-6100 cells in RAW 264.7 and 300-3470 cells in IPMΦ per well are presented as mean ± SEM. #$p < 0.05$ LPS; vs. LPS+A_{2A}R agonist treated cells.

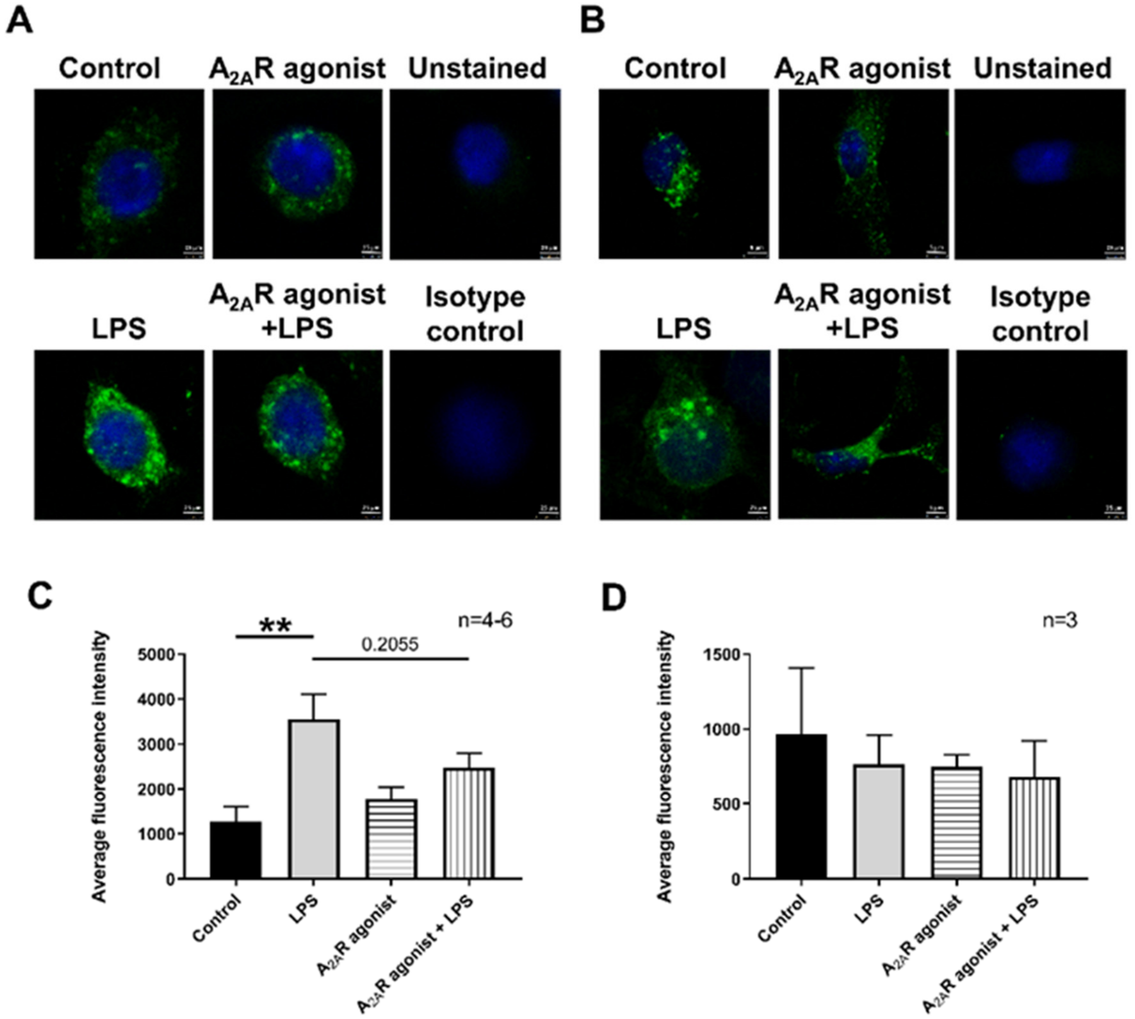

Figure S2. A_{2A}R activation decreases cell surface deposition of LAMP2 in macrophages. Immunofluorescence staining of (A) RAW 264.7 cells and (B) IPMΦ cells were made using LAMP2 specific, Alexa-488 conjugated antibody (green). Nuclei of macrophages were stained with DAPI (blue). Microscopic images were made with Leica SP8 Confocal microscope. LAMP2 specific fluorescence intensity was measured in (C) RAW264.7 cells and (D) IPMΦ cells after LPS activation and treatment with the A_{2A}R agonist CGS21680 by iCys Laser Scanning Cytometry. Statistical analysis based on 3-6 independent experiments, in each experiments 500-2500 cells were calculated and are presented as mean ± SEM. $^{**}p < 0.01$ control vs. LPS treated cells and $p = 0.205$ A_{2A}R agonist +LPS vs. LPS treated cells.

Adenosine A_{2A} receptor activation regulates Niemann-Pick C1 expression and localization in macrophages

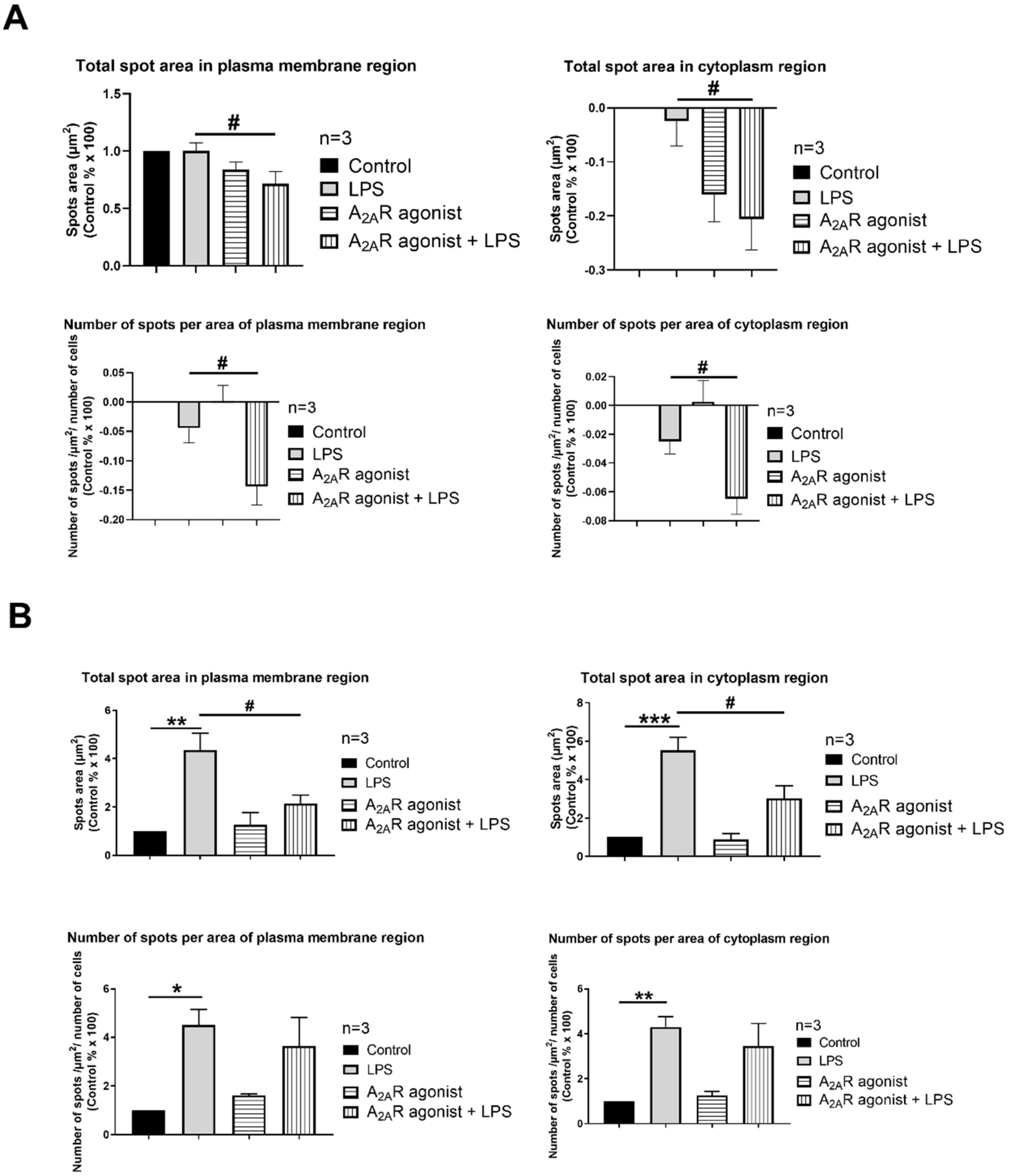

Figure S3. $A_{2A}R$ activation decreases cell surface deposition of LAMP2 in RAW 264.7 and mouse IPMΦs cells. Immunofluorescence staining of RAW 264.7 (A) and IPMΦ cells (B) were made using LAMP2 specific, Alexa-488 conjugated antibody. LAMP2 specific fluorescence intensity was measured after LPS activation and treatment with the $A_{2A}R$ agonist CGS21680. Cellular features such as total spots area and number of spots per area of membrane and cytoplasm regions were extracted. Data obtained from the individual analysis of 550-5450 cells in RAW 264.7 and 500-1350 cells in IPMΦ per well are presented as mean ± SEM. * $p < 0.05$; ** $p < 0.01$; ***$p < 0.001$ control (vehicle-treated) vs. LPS activated cells and #$p < 0.05$ LPS vs. LPS+$A_{2A}R$ agonist-treated cells.

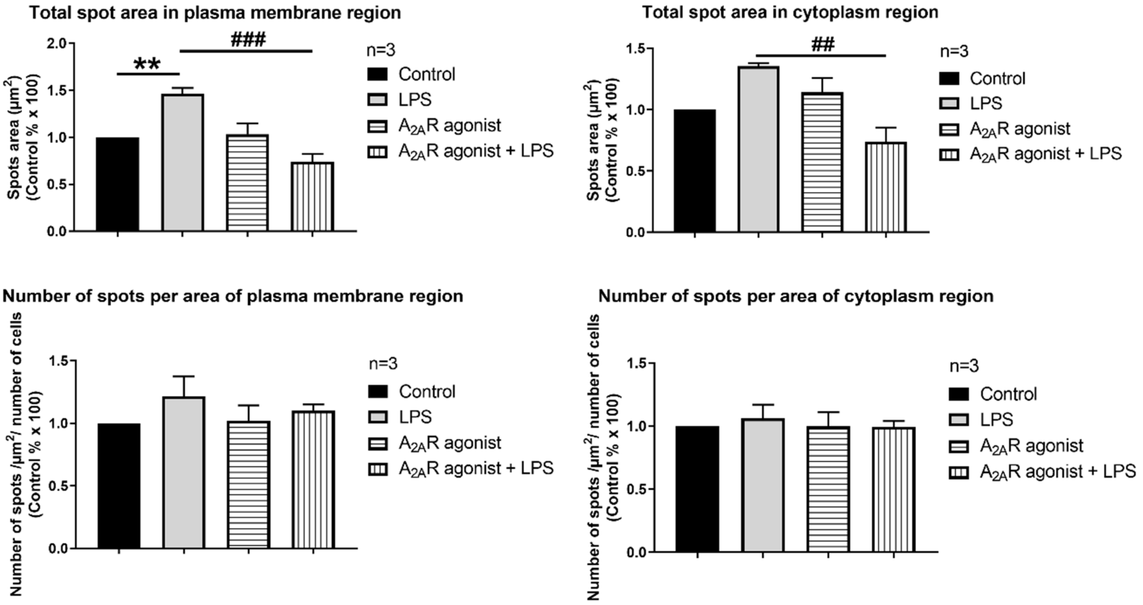

Figure S4. $A_{2A}R$ activation decreases Early Endosome A1 (EEA1) expression in mouse IPMΦs. Immunofluorescence staining of IPMΦ cells was made using EEA1 specific primary and Alexa-488 conjugated anti-rabbit secondary antibody. EEA1 specific fluorescence intensity was measured after LPS activation and treatment with the $A_{2A}R$ agonist CGS21680. Cellular features such as total spots area and number of spots per area of membrane and cytoplasm regions were extracted. Data obtained from the individual analysis of 370-3445 different cells are presented as mean ± SEM. ** $p < 0.01$ control (vehicle-treated) vs. LPS activated cells and ## $p < 0.01$; ### $p < 0.001$ LPS vs. LPS+$A_{2A}R$ agonist-treated cells.

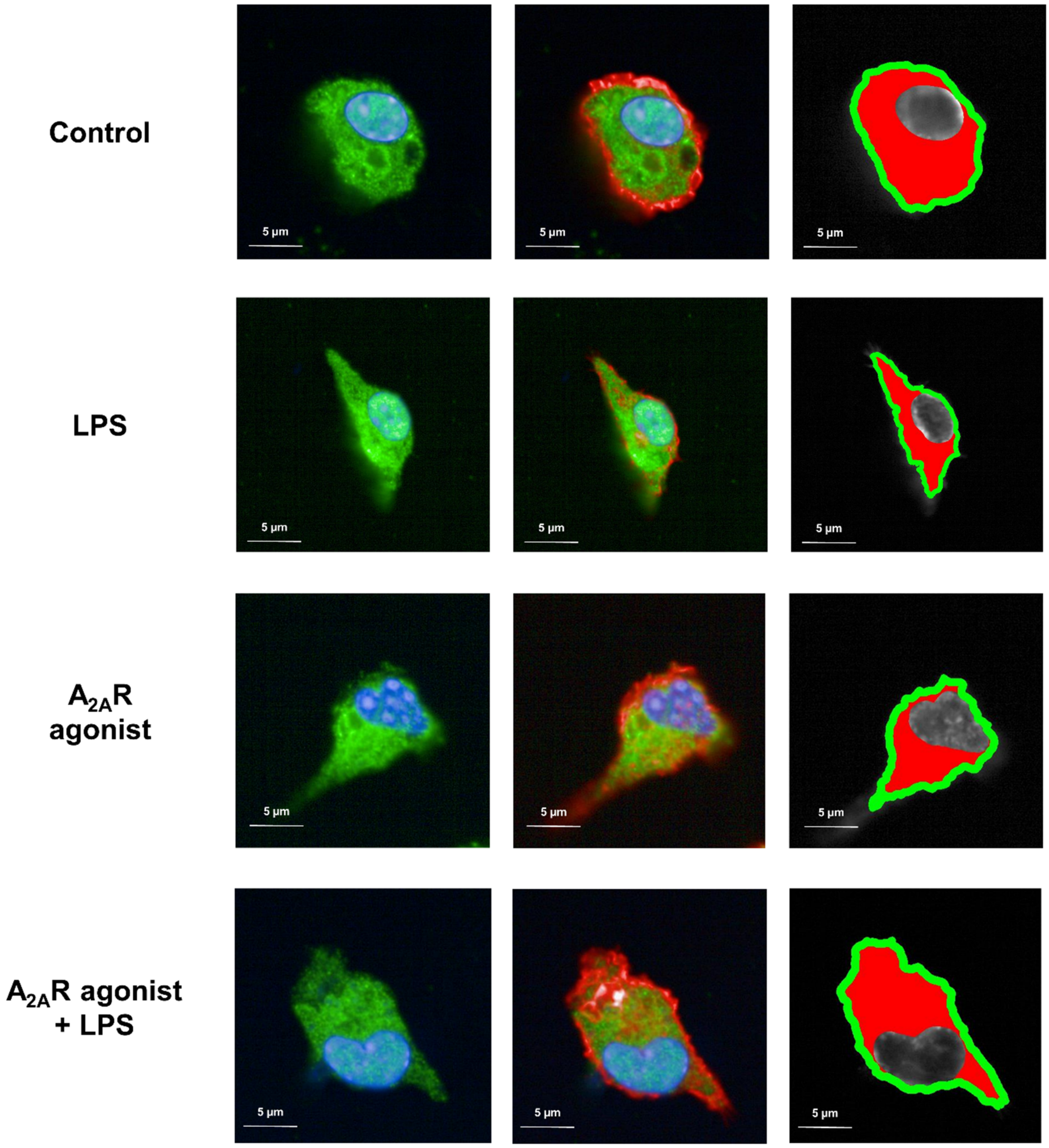

Figure S5. Immunofluorescence staining of mouse IPMΦ cells was made using NPC1 specific, primary and Alexa-488 conjugated anti-rabbit secondary antibody (green). Nuclei of macrophages were stained with DAPI (blue) and F-actin was visualized by Texas Red-X Phalloidin (red). Microscopic images show the cytoplasm (red) and plasma membrane (green) regions identified by Harmony4.8 program.

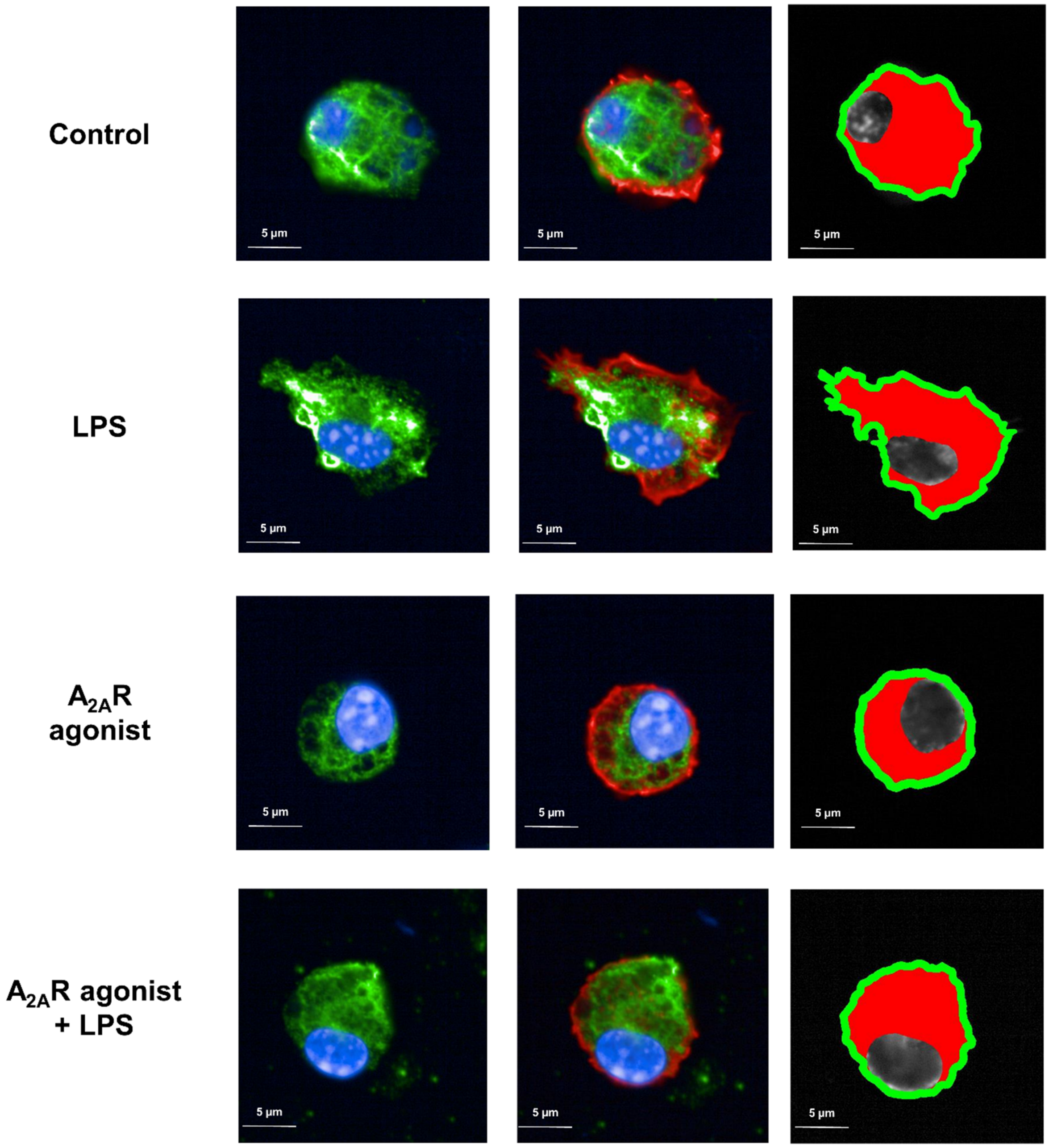

Figure S6. Immunofluorescence staining of mouse IPMΦ cells was made using EEA1 specific, primary and Alexa-488 conjugated anti-rabbit secondary antibody (green). Nuclei of macrophages were stained with DAPI (blue) and F-actin was visualized by Texas Red-X Phalloidin (red). Microscopic images show the cytoplasm (red) and plasma membrane (green) regions identified by Harmony4.8 program.
